# OLD AND FORGOTTEN? CARE FOR ELDERS IN MEXICO AND THE U.S.

**DOI:** 10.1093/geroni/igz038.249

**Published:** 2019-11-08

**Authors:** Emma Aguila, Jaqueline L Angel, Kyriakos Markides

**Affiliations:** 1 University of Southern California, Los Angeles, California, United States; 2 The University of Texas at Austin, Austin, Texas, United States; 3 University of Texas Medical Branch, Galveston, Texas, United States

## Abstract

The United States and Mexico differ greatly in the organization and financing of their old-age welfare states. They also differ politically and organizationally in government response at all levels to the needs of low-income and frail citizens. While both countries are aging rapidly, Mexico faces more serious challenges in old-age support that arise from a less developed old-age welfare state and economy. For Mexico, financial support and medical care for older low-income citizens are universal rights, however, limited fiscal resources for a large low-income population create inevitable competition among the old and the young alike. Although the United States has a more developed economy and well-developed Social Security and health care financing systems for the elderly, older Mexican-origin individuals in the U.S. do not necessarily benefit fully from these programs. These institutional and financial problems to aging are compounded in both countries by longer life spans, smaller families, as well as changing gender roles and cultural norms. In this interdisciplinary panel, the authors of five papers deal with the following topics: (1) an analysis of old age health and dependency conditions, the supply of aging and disability services, and related norms and policies, including the role of the government and the private sector; (2) a binational comparison of federal safety net programs for low-income elderly in U.S. and Mexico; (3) when strangers become family: the role of civil society in addressing the needs of aging populations; and (4) unmet needs for dementia care for Latinos in the Hispanic-EPESE.

